# Hepatic epithelioid Hemangioendothelioma presenting synchronously with hepatocellular carcinoma-a case report

**DOI:** 10.1093/omcr/omae140

**Published:** 2024-11-25

**Authors:** Anna Pan, Shuang Xu, Liang Hong, Wenwei Zhu, Yu Zhou, Xiaoyang Wang

**Affiliations:** Department of Infectious Diseases, The Third Affiliated Hospital of Wenzhou Medical University, Wenzhou, Zhejiang, China; Department of Infectious Diseases, The Third Affiliated Hospital of Wenzhou Medical University, Wenzhou, Zhejiang, China; Department of Infectious Diseases, The Third Affiliated Hospital of Wenzhou Medical University, Wenzhou, Zhejiang, China; Department of General Surgery, Huashan Hospital Affiliated to Fudan University, Shanghai, China; Department of Infectious Diseases, The Third Affiliated Hospital of Wenzhou Medical University, Wenzhou, Zhejiang, China; Department of Radiology, The Third Affiliated Hospital of Wenzhou Medical University, Wenzhou, Zhejiang, China

**Keywords:** hepatic epithelioid hemangioendothelioma, hepatocellular carcinoma

## Abstract

Backgrounds: Primary hepatic epithelioid hemangioendothelioma (HEHE) is a rare neoplasm of vascular origin with varying biologic behavior, making it challenging to diagnose.

Case presentation: We present a case of synchronous hepatocellular carcinoma (HCC) and HEHE in a 43-year-old Chinese male patient. Multiple hypoechoic liver lesions were depicted, but no specific imaging findings were detected on enhanced computed tomography (CT) or contrast-enhanced magnetic resonance imaging (MRI). The patient then underwent [^18^F]-FDG PET/CT, [^11^C]-acetate PET/CT, and [^68^Ga]Ga-FAPI-04 PET/CT. The HEHE lesions demonstrated no uptake on both ^18^F-FDG and ^11^C-acetate PET/CT imaging, but presented a clear visualization in [^68^Ga]Ga-FAPI-04 PET/CT. The largest lesion located in segment VII was finally diagnosed as HCC, while the other smaller ones were diagnosed as HEHE, which was confirmed by immunohistochemical staining for CD31. To the best of our knowledge, only 2 cases have been reported in the worldwide literature, and the first case undertook both ^11^C-acetate and [^68^Ga]Ga-FAPI-04 PET/CT instead of ^18^F-FDG PET/CT.

Conclusion: In this report, we show that HCC and HEHE may occur synchronously, and HEHE should be considered when liver lesions are detected. [^68^Ga]Ga-FAPI-04 PET/CT has great potential in the detection, staging and therapy selection of HEHE.

## Introduction

HEHE is a rare vascular neoplasm with low-grade malignancy potential. Previous reports have described the difficulty of diagnosing this rare tumor due to its variable clinical course and imaging appearance. Herein, we present a case of synchronous HCC and HEHE, which has only reported twice in the world wide literature to our best knowledge. One case involves a 58-year-old male who was found to have elevated GT levels in his blood. Further imaging revealed multiple liver and lung lesions. A biopsy confirmed that the liver lesions were HEHE with lung metastasis, while the left lobe liver mass, which was removed via laparoscopic surgery, was ultimately diagnosed as HCC upon pathology [1]. The other case is an 84-year-old elderly male who visited the hospital due to back pain. A CT scan incidentally discovered a liver mass. Imaging suggested that segment 3 of the liver had cholangiocarcinoma, and segment 2 had a solitary HCC. The pathology report confirmed that the mass in segment 3 was HEHE, and the tumor in segment 2 was a primary liver cancer [2]. While multiple liver lesions are detected, HEHE should be taken into consideration. [^68^Ga]Ga-FAPI-04 PET/CT has great potential for detecting HEHE.

## Case presentation

In December 2020, a 43-year-old man with a 9-year-history of hepatitis B underwent an ultrasound scan during a routine medical examination in Ruian People’s Hospital, which revealed several hypoechoic lesions in his liver.

**Figure 1:**
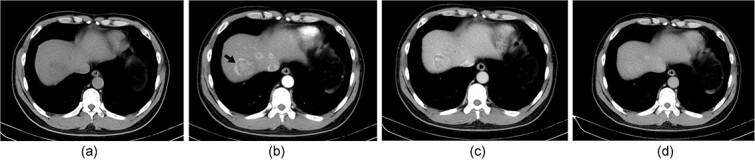
(a) The lesions exhibit low density with unclear margins on non-contrast computed tomography (CT) imaging. (b-d) on dynamic contrast-enhanced CT imaging, centripetal enhancement can be observed. The sizes of lesions varied, with the largest being 4.1 × 4.3 cm in diameter (black arrow), with irregular shape.

Laboratory tests showed a slight elevation in alpha-fetoprotein (AFP) levels (AFP: 32.9 ng/ml) while other tumor markers such as carbohydrate antigens 19–9, 125, and 153, and carcinoembryonic antigen were within normal limits. The lesions appeared as low density with unclear margins on plain computed tomography (CT) imaging, and showed centripetal enhancement from the arterial to the portal phase on dynamic contrast-enhanced CT imaging. The sizes of the lesions varied, with the largest being an irregularly shaped lesion measuring 4.1 × 4.3 cm in diameter (Fig. 1).

**Figure 2:**
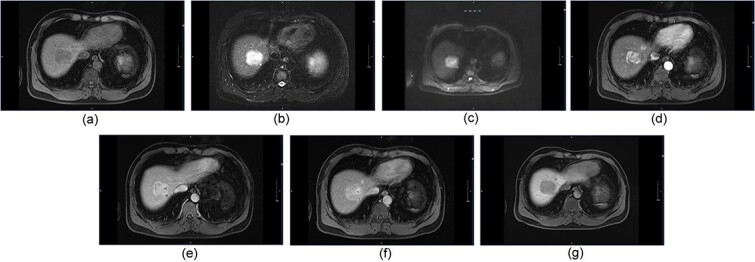
(a) On T1-weighted imaging (T1WI), the lesions exhibit low signal intensity, similar to that on non-contrast computed tomography (CT). On T2-weighted imaging (T2WI). (b) and diffusion-weighted imaging (DWI). (c) the lesions show heterogeneous high signal intensity relative to the liver parenchyma. (d-g) on dynamic contrast-enhanced MRI scans, the largest lesion shows immediate peripheral ring-like enhancement with progressive continuous enhancement with a central enhancing scar. Other smaller ones show ring-like enhancement, with gradual centripetal enhancement on the portal venous and delayed phases.

On dynamic contrast-enhanced magnetic resonance imaging (MRI), the largest lesion exhibited immediate peripheral ring-like enhancement, followed by progressive continuous enhancement and a central enhancing scar. Meanwhile, smaller lesions exhibited ring-like enhancement with gradual centripetal enhancement during the portal venous and delayed phases (Fig. 2). For further examination, the patient was referred to Shanghai Huashan Hospital on December 30th, 2020.

**Figure 3:**
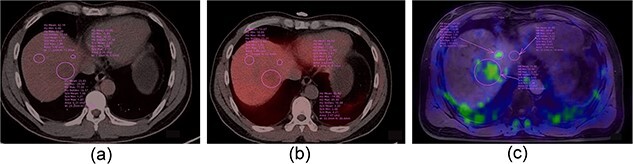
(a) [^18^F]-FDG PET/CT scan showing no obvious uptake. (b) [^11^C]-acetate PET/CT scan indicating slight uptake only for the largest lesion located in lobe VII (SUV_max_ = 4.21, TBR = 1.45), whereas other smaller ones show no uptake. (c) ^68^Ga-FAPI PET/CT scan not only shows strong, high uptake for the largest lesion (SUV_max_ = 1.94 TBR = 3.08), but also the smaller intrahepatic lesions (SUV_max_ = 1.47, TBR = 2.33). SUV_max_, maximum standardized uptake value; TBR, tumor-to-background ratio.

The patient promptly underwent [^18^F]-FDG positron emission tomography (PET)-CT, which revealed no obvious uptake. However, [^11^C]-acetate PET/CT indicated slight uptake only for the largest lesion located in lobe VII (maximum standardized uptake value [SUV_max_] = 4.21, tumour-to-background ratio (TBR) = 1.45), whereas other smaller ones exhibited no uptake. Interestingly, [^68^Ga] Ga-FAPI-04 PET/CT scan not only showed strong, high uptake for the largest lesion (SUV_max_ = 1.94, TBR = 3.08), but also for the smaller intrahepatic lesions (SUV_max_ = 1.47, TBR = 2.33) (Fig. 3).

Based on the existing examinations, a preliminary diagnosis of hepatocellular carcinoma (HCC) was made. The patient then received partial hepatectomy in 6 days, and the diagnosis was confirmed by pathological analysis. The largest lesion located in segment VII was identified as a solitary HCC, while the other smaller lesions were diagnosed as hepatic epithelioid hemangioendothelioma (HEHE).

**Figure 4:**
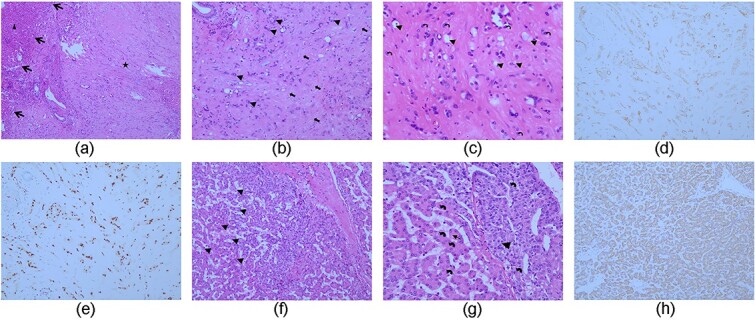
(a) The tumor lesion showed an ill- defined border, and fibrotic and paucicellular changes with residual hepatic lobules. (HE staining, ×40) (star: The tumor; long arrows: Ill- defined border; arrowhead: Hepatic lobules) (b) tumor cells were epitheloid or mild elongated in appearance and scattered in small clusters and strings surrounded by a fibrous stroma. (HE staining, ×100) (arrowhead: Small tumor clusters; thick arrows: Fibrous stroma) (c) tumor cytoplasm was clear and mild eosinophilic, some of which showed a vacuole containing erythrocytes. (HE staining, ×200) (crooked arrows: Small nucleoli; arrowhead: Intracytoplasmic vacuoles) (d，e) diffuse positive for CD31 and ERG1. (f) Was cellular and mimicked a thin trabecular plate architecture of liver. Vascular invasion could be identified at left. (HE staining, ×100) (arrowhead: Tumor cell trabecula; thick arrows: Vascular invasion). (G) tumor cell trabecula varied by 2 cells or greater in thickness. These cells were polygonal and showed round vesicular nuclei, prominent nucleioli, and abundant cytoplasm present as oxyphilic to slightly basophilic. (HE staining, ×200) (crooked arrows: Prominent nucleioli; long arrowhead: Oxyphilic cytoplasm; arrowhead: Slightly basophilic cytoplasm) (h) was positive for hep1.

Microscopically, smaller lesions appeared as nests and cords of epithelioid endothelial cells, with intracytoplasmic lumina spread throughout the myxohyaline stroma. CD31 and ERG1 staining was strongly positive, confirming the diagnosis of HEHE (Fig. 4A–E). The segment VII hepatic lesion exhibited Hep1 positivity on immunohistochemistry, confirming the diagnosis of HCC (Fig. 4F–H).

The patient was discharged on postoperative day 9 and no recurrence has been detected during his follow-up until now.

## Discussion

HEHE is a rare neoplasm of vascular origin, with an incidence of less than 0.1 cases per 100 000 people [3]. The etiology of HEHE remains unclear. Several possible pathogenic and risk factors have been identified, including exposure to chloroethylene, polyurethane, or silica; oral contraceptive use; primary biliary cirrhosis; viral hepatitis; exposure to asbestos; and alcohol use [4]. Females are more commonly affected, with a 3:2 predominance compared to males [5].

In non-contrast CT scans, tumors usually appear as low-density lesions, preferentially involving the subcapsular region [6]. On MRI, lesions are usually hypointense on T1-weighted images and hyperintense on T2 [7]. Following contrast agent administration, lesions may demonstrate only immediate mild peripheral rim-like enhancement followed by centripetal filling, or a lack of enhancement may be observed on dynamic post-contrast imaging, depending on the degree of fibrosis, myxoid, and hyalinized components.

HEHE may demonstrate distinctive imaging features such as the lollipop sign, target-like sign, capsular retraction, subcapsular positioning, and a propensity for coalescence into large confluent masses. However, these characteristic findings are not commonly presented by most patients, which contributes to the challenge in radiological diagnosis [6]. In the present case, none of the aforementioned imaging features were observed. It was initially misdiagnosed as a HCC with intrahepatic metastasis.

To better discriminate intrahepatic lesions, ^18^F-FDG PET/CT has been widely used in clinical practice for decades. Well in line with the findings of previous studies, HCC lesions with moderate differentiation located in lobe VII showed no FDG uptake, slight ^11^C-acetate uptake, and remarkable [^68^Ga] Ga-FAPI uptake. Several studies have reported the performance of ^18^F-FDG PET/CT for detecting HEHE. Most HEHE lesions demonstrate a slight increase in the uptake of 18F-FDG, whereas approximately one-third of HEHE lesions show similar uptake in the liver parenchyma [8]. Kitapci et al. emphasized that dual-time-point PET/CT acquisition can facilitate the detection of HEHE, which may not be visible on routine 60-min post-injection examination [9].Owing to the rarity of the malignancy, no previous report on 11C-acetate and [^68^Ga] Ga-FAPI-04 PET/CT for detecting HEHE has been published to date, to the best of our knowledge. In the present case, the HEHE lesions demonstrated no uptake in either 18F-FDG or ^11^C-acetate PET/CT imaging, but presented a clear visualization on [^68^Ga] Ga-FAPI-04 PET/CT. This indicates that [^68^Ga] Ga-FAPI is a promising new tracer for the detection of HEHE.

Due to the rarity of HEHE, no randomized controlled trials with multiple treatment strategies have yet been performed, and optimal treatment strategies for this tumor have not been established [4]. Hepatic resection is considered the first choice of treatment, especially for the single-nodular type. Liver transplantation is the ultimate treatment for multifocal, diffuse, non-resectable, or recurrent tumors [10]. Our patient underwent partial hepatectomy, and no recurrence was detected during follow-up.

In conclusion, HEHE is a rare malignant tumor. To the best of our knowledge, this is the third reported case of synchronous HCC and HEHE, and the first case involving both ^11^C-acetate and [^68^Ga] Ga-FAPI-04 PET/CT, in addition to ^18^F-FDG PET/CT. Our clinical data demonstrate the outstanding capability of [^68^Ga] Ga-FAPI-04 PET/CT to visualize both HCC and HEHE. [^68^Ga] Ga-FAPI-04 PET/CT together with [^11^C]-acetate PET/CT has great potential for differentiating intrahepatic lesions. Our case also highlights the great potential of [^68^Ga] Ga-FAPI-04 PET/CT for the detection, staging, and therapeutic selection of HEHE. In the future, more cohort studies can be designed to investigate the ability of [68Ga] Ga-FAPI-04 PET/CT to detect and differentiate HEHE from other liver pathologies, such as Hepatocellular Carcinoma (HCC), as well as its accuracy and reliability in diagnosing HEHE at various stages.

## Data Availability

All data and materials generated or analyzed during this study are included in this article. Further information can be obtained from the corresponding author on reasonable request.
